# Inter-disciplinary management of a patient with severely attrited teeth

**DOI:** 10.4103/0972-124X.75916

**Published:** 2010

**Authors:** Shyam Padmanabhan, Venkateswara Allu Reddy

**Affiliations:** *Department of Periodontics, Vydehi Institute of Dental Sciences and Research Centre, Bangalore, India*; 1 *Department of Prosthodontics, Vydehi Institute of Dental Sciences and Research Centre, Bangalore, India*

**Keywords:** Anterior dental esthetics, anterior guidance, anterior tooth wear, bruxism, crown-lengthening, Inter-disciplinary dentistry, occlusion

## Abstract

With increased awareness about dental esthetics, multidisciplinary periodontal therapy has begun to gain momentum. Management of severely attrited teeth is a challenging situation and is dealt with a multidisciplinary approach. In cases of severe tooth wear, the crown height is drastically reduced, in some cases up to the gingival level. This might require a contribution from the disciplines of endodontics, periodontics, orthodontics and prosthodontics for predictable results. Herein we describe the management of one such case.

## INTRODUCTION

Dentistry in general and esthetic dentistry in particular can greatly benefit from assuming an Inter-disciplinary perspective. With the increasing popularity of esthetic dentistry, an understanding of the therapeutic synergies brought about by an Inter-disciplinary approach has developed. As a result, crown-lengthening procedures have become an integral component of the esthetic armamentarium and are utilized with increasing frequency to enhance the appearance of restorations placed within the esthetic zone. Recent studies have concluded that very few general dentists are happy to carry out crown-lengthening. Therefore, integrated, Inter-disciplinary treatment (periodontal surgery; endodontic, restorative and prosthetic treatment; and improvement of occlusion) is required.

## CASE REPORT

A male patient aged 47 years presented with a chief complaint of worn-out lower front teeth. A complete oral examination followed by a detailed medical and dental history was done and recorded. The oral hygiene status of the individual was fair [Oral hygiene index (OHI) score=1.5]. Medical history revealed that the patient was a known diabetic under medication Glipizide once daily (OD). He presented with a history of occasional grinding of teeth.

**Figure 1:**
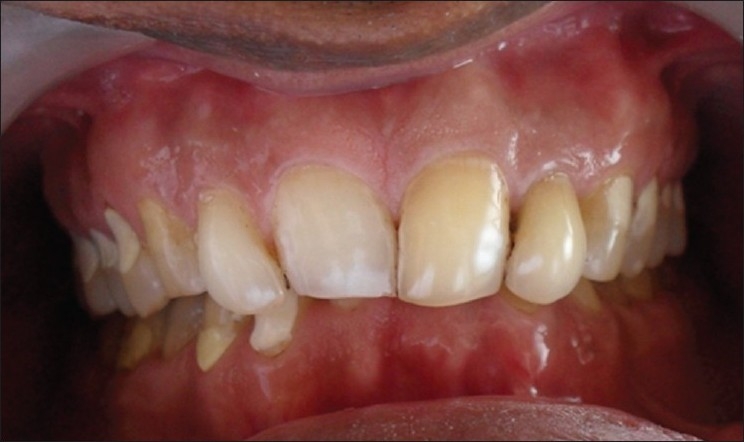
Pre-treatment

**Figure 2:**
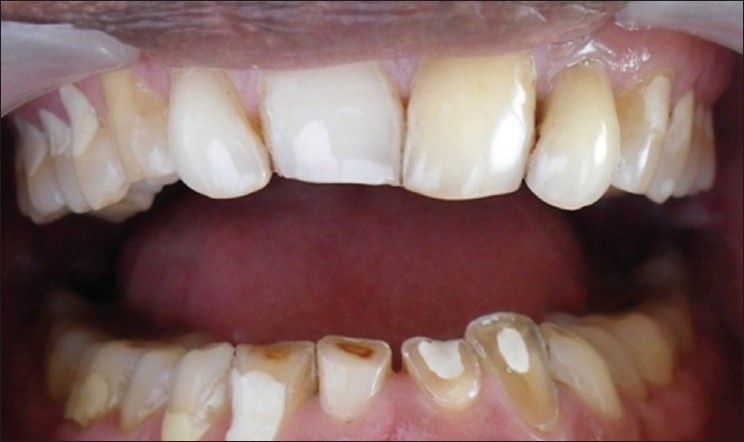
Endodontically treated 21, 22, 32 and 33

Intraoral examination revealed, congenitally missing teeth in relation to 31, 41. All the remaining teeth showed signs of attrition, without any specific symptoms. Most of the posterior teeth showed class V fillings, which suggests that the lesions could be due to stress caused by the parafunctional habits like bruxism and clenching. Lower anterior teeth in relation to 32, 33, 42 and 43 were severely attrited with inadequate clinical crown length. Teeth numbers 11, 12, 21 and 22 were slightly palatally tilted, and the palatal aspects of these teeth also showed gross attrition, due to deep bite. Teeth numbers 21, 22, 32 and 33 were already endodontically treated as he had a history of pain and recurrent infections in relation to these teeth [Figures 1 and 2].

Evaluation of vertical dimension of occlusion did not reveal any signs of loss of vertical dimension of occlusion (VDO). Occlusal analysis was done intraorally, and it revealed that there was no definite anterior guidance; and, thereby, absence of any mutually protected occlusion. Lateral movements of mandible showed that on the left working side, there was group function; and on the right nonworking side, there were interferences in the molar regions (46, 47 and 48). On the right working side, there were canine and premolar occlusions, with nonworking side on the left showing interferences in the second and third molars (37 and 38). Even with these clinical signs, the patient did not present with any symptoms of Tempero Mandibular Joint (TMJ) dysfunction or disorder.

The treatment was planned in three phases.

### Phase I therapy — Preventive

- Scaling and root planing

### Phase II therapy — Surgical and restorative

- Crown-lengthening (in relation to lower anterior teeth)
- Porcelain-fused-to-metal (PFM) crowns (in relation to 11, 12, 21, 22; and 32, 33, 42, 43)

### Phase III — Maintenance

- Follow-up and oral hygiene instructions
- Use of a night guard to minimize effects of bruxism and also to act as a habit-breaking appliance

First, the patient’s maxillary and mandibular impressions were made with irreversible hydrocolloid impression material, and two pairs of diagnostic casts were prepared.

A complete oral prophylaxis, including root planing, was done as a part of initial therapy. The patient was recalled after a week for evaluation and was found to maintain satisfactory oral hygiene.

### Periodontal management — Crown-lengthening

The pre-surgical analysis consisted of the following[1]

1. Determination of the finish line prior to surgery[1]
2. Transcrevicular circumferential probing prior to surgery, to establish the biologic width.

Prior to the procedure, evaluation of the tooth and periodontium was done clinically and radiographically. The clinical measurements included probing sulcus depth, biologic width, osseous crest, pulpal involvement, gingival health, loss of mesial/distal occlusal space, anticipated final margin placement.

Radiographic assessment included level of alveolar crest, pulpal involvement, root length, root form, crown-to-root ratio (pre-treatment or post-treatment).

There was a generalized mild loss of attachment (1-2 mm). The crown and root lengths were evaluated from the radiographs. The findings were as follows:

- Crown length was 6 mm, 6 mm, 5 mm and 5 mm in relation to 42, 43, 32 and 33, respectively.
- Root length was 12 mm, 14 mm, 10 mm and 11 mm in relation to 42, 43, 32 and 33, respectively.

The amount of crown-lengthening planned was about 2 mm more than presently available crown length. Anticipated crown-root ratio following completion of the procedure, on an average, would be 1:1.2, which is considered favorable.

The patient was scheduled for an apically repositioned flap to provide adequate clinical crown length to facilitate prosthesis.

**Figure 3:**
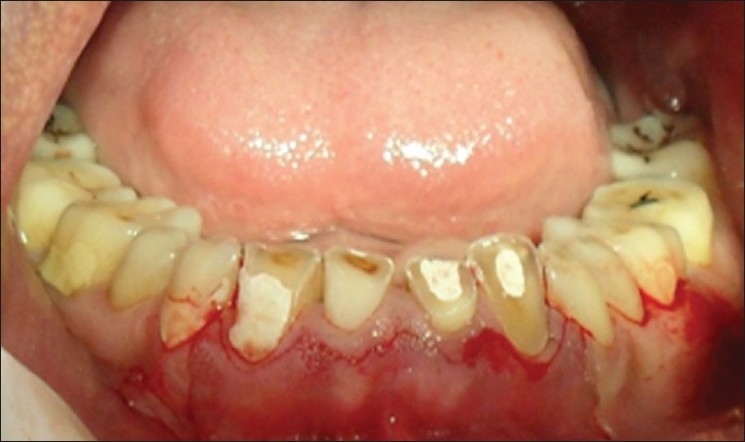
Inverse bevel incisions placed

**Figure 4:**
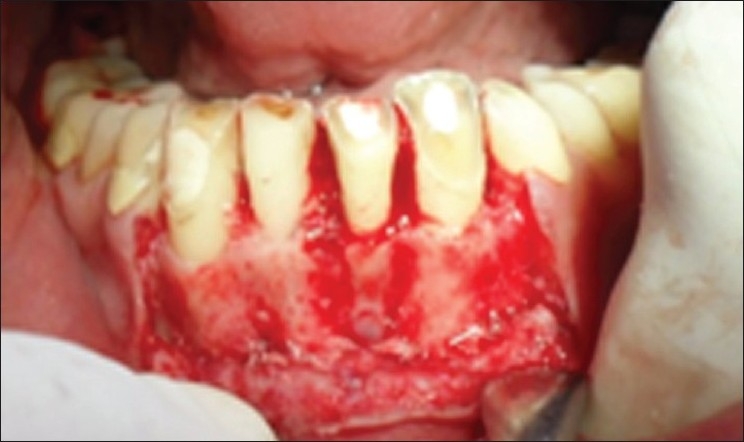
Flap reflected and ostectomy done

Inverse bevel incisions were placed to reduce the bulky tissue. The flaps were extended one tooth distal of both the lower canines to permit adequate access to perform osseous surgery. Maximum preservation of keratinized gingiva was recommended. The scalloping of the flap was performed anticipating the final underlying osseous contour. A full-thickness flap was reflected up to the mucogingival junction and split apically [Figures 3 and 4].

Following proper degranulation, osteoplasty followed by ostectomy was performed to obtain at least 4 mm of healthy tooth structure above the alveolar crest.

Ostectomy and scalloping of the bone buccally and lingually were performed not only on the affected tooth but also onto the adjacent teeth for blending and gradulization of osseous architecture.

**Figure 5:**
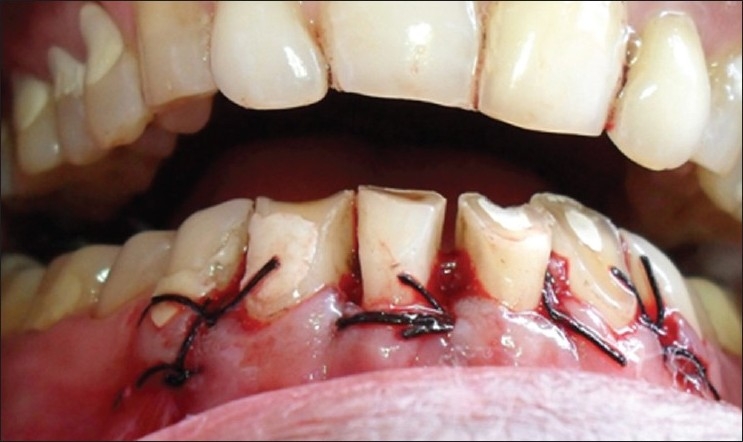
Sutures placed and flap repositioned at the crest of alveolar bone

Since the amount of keratinized gingiva was around 3 to 4 mm, the flap was positioned at the crest of alveolar bone and sutured using a black braided silk suture [Figure 5]. Routine postoperative instructions were given. The medications prescribed were, amoxicillin 500 mg tid for 5 days and Paracetamol qid for 3 days.

**Figure 6:**
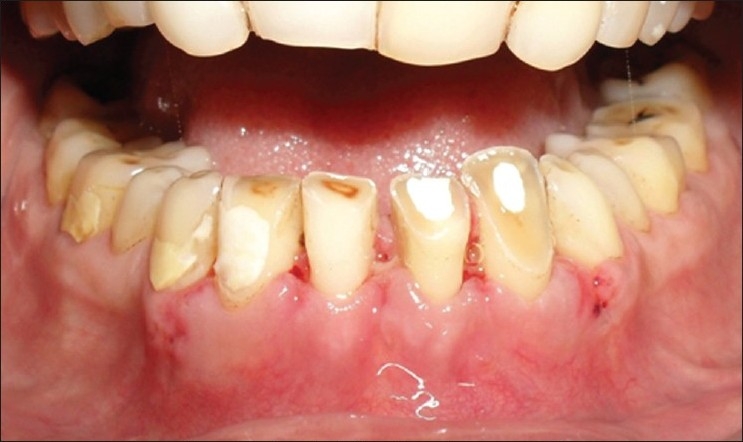
Surgical site after suture removal

The patient was recalled after 1 week for suture removal [Figure 6], following which the patient was referred to the Department of Prosthodontics for the fabrication of PFM crowns in relation to 11, 12, 21, 22, 32, 33, 42 and 43.

### Prosthodontic management — Esthetic and occlusal rehabilitation

The main aim of this treatment was to create esthetic and functional crowns and thereby develop mutually protected occlusion by generating an anterior guidance to disocclude posteriors, thereby generating group function/canine and premolar occlusion on working side and removing nonworking side interferences.

Anterior-bite plane splint was fabricated with a self-cure acrylic resin to disocclude the posterior teeth in centric and eccentric jaw positions, so as to allow for the passive supraeruption of all the posterior teeth. Only the lingual surfaces and incisal edges of lower anteriors were covered with the bite plane. splint Labial surface was kept open so that the post-surgical maintenance in the region of the surgery was not hampered. The patient was instructed to wear the splint through the day for at least4 to 6 weeks, so as to allow for passive supraeruption of posterior teeth.

The patient was recalled after a month and evaluation of occlusion was done. It was observed that there was no significant supraeruption of posteriors, but there was a good amount of tooth exposed in relation to 32, 33, 42 and 43 for the abutment height post-surgery. Therefore, teeth preparation to receive PFM crowns was scheduled in relation to 11, 12, 21, 22, 32, 33, 42 and 43.

**Figure 7:**
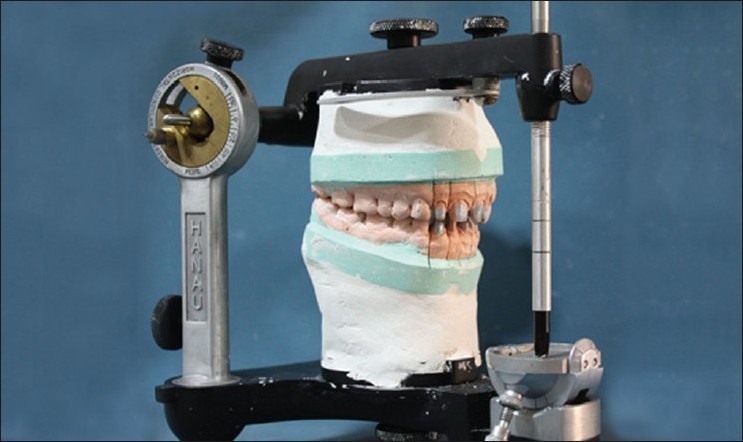
Casts mounted on a Hanau articulator

Upper and lower elastomeric impressions (3M ESPE – Express XT putty soft and light body) were made for the fabrication of final definitive restorations. Provisional restorations for both upper and lower teeth were fabricated with self-polymerizing acrylic resin (DPI – self-cure – tooth-molding powder). Protrusive wax record was taken. Provisional restorations were cemented with non-eugenol temporary cement (3M ESPE – Relyx Temp lute). Casts were made from the elastomeric impressions and mounted on semi-adjustable articulator (Hanau, non-arcon) with the help of a face bow and interocclusal records [Figure 7].

With the help of protrusive record, horizontal condylar guidance angle was generated, which was 45°. Lateral condylar guidance angle was calculated based on the Hanau formula, L = (H/8) + 12, which was found to be 17°. Articulator was programmed according to these values.

**Figure 8:**
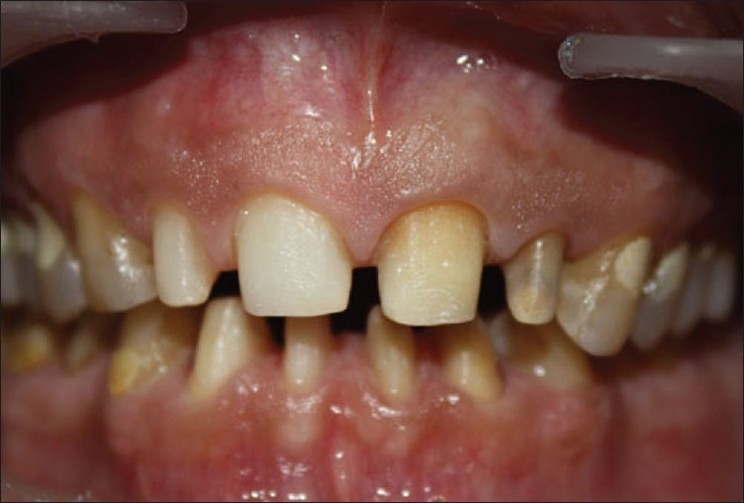
Teeth prepared for PFM crowns

**Figure 9:**
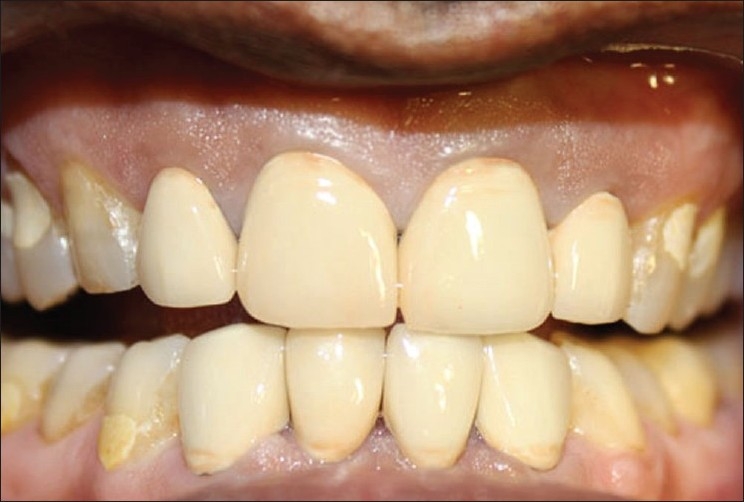
PFM crowns placed

Wax patterns for the fabrication of PFM restorations were made and cast with metal. In case of upper incisors, labial ceramic facing with palatal metal backing was planned; whereas for the lower anteriors, full ceramic coverage was planned. In the final crowns, overjet was slightly increased to compensate for the deep bite. Metal try-in was done to check the fit of the crowns on the natural teeth. It also gives an idea about overjet and overbite. After the fabrication of PFM crowns, refining of occlusion was done in the patient intraorally [Figures 8 and 9].

**Figures 10, 11 and 12:**
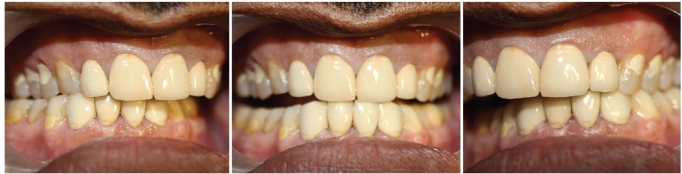
Occlusion evaluated and adjusted

Care was taken so that the occlusion on the working side was not canine guided, as canine is restored with a crown and if the habitual grinding continues, it can lead to ceramic fracture in relation to the canine 33, 43. Therefore, if canine is disoccluding the premolars and molars on the working side, the length of 33, 43 is so adjusted that there is group function / canine and premolar guidance. On the left side, group function; and on the right side, canine and premolar guided occlusion were generated (on the working sides) as planned. All the non working interferences on both the sides were removed [Figures 10, 11 and 12].

**Figure 13:**
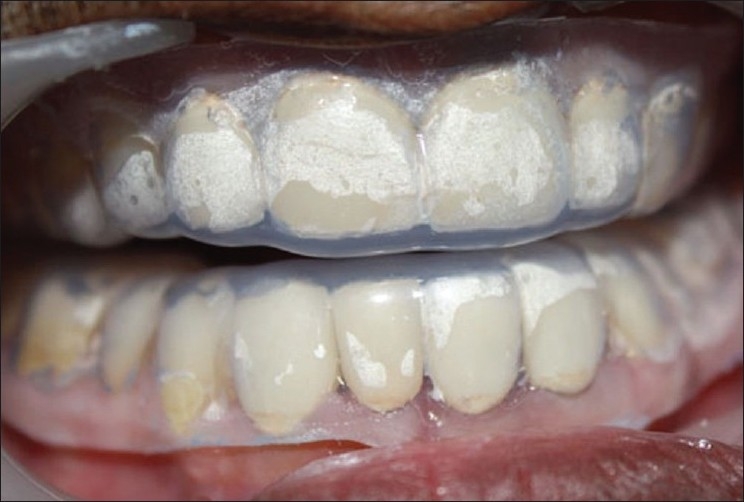
Night guard in place

**Figure 14:**
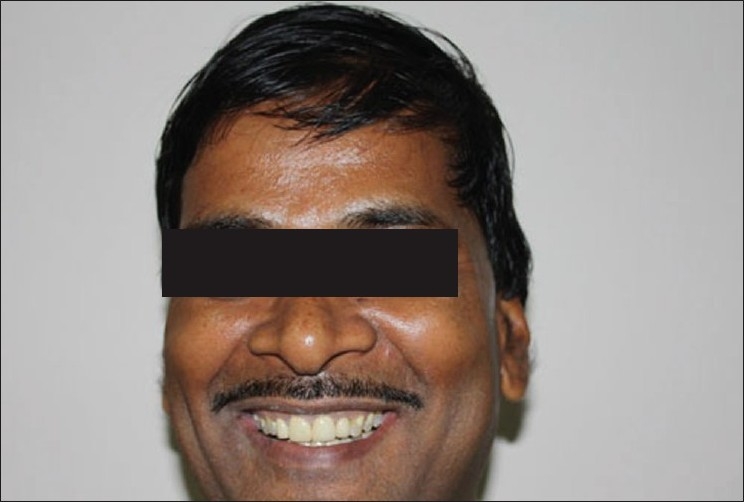
Postoperative facial photograph of patient

After refining the occlusion, the crowns were glazed and cemented with glass ionomer cement (3M ESPE -Easy mix). After cementation, alginate impressions of upper and lower arch were made, and casts obtained were used for the fabrication of night guard. Both upper and lower night guards were made and dispensed to the patient with instruction to wear them every night, so as to break the habit of grinding and at the same time protecting the crowns from fracture [Figures 13 and 14].

## DISCUSSION

D. W. Cohen[2] in 1962 introduced the concept of crown-lengthening, which is presently a procedure that often involves a combination of soft tissue reduction/ removal, osseous surgery and/ or orthodontic treatment for tooth exposure. To ensure a good marginal seal with retention for both provisional and final restorations, the amount of tooth structure that is exposed above the osseous crest must be about 4 mm, i.e., enough to provide for a stable dentogingival complex and biologic width to permit proper tooth preparation and account for an adequate margin placement.[3–7]

Kois[8] has stated that 3 mm is necessary to satisfy the requirements for a stable biologic width (2.04 mm, biologic width; 1 mm, sulcus depth). Violation of the biologic width may result in inflammation and bone resorption.

Full-mouth rehabilitation with crowns on all the teeth was not considered, as there was no significant tooth wear of the posterior teeth and also there was no loss of VDO. So crowns were planned only for the lower anteriors and upper incisors. But since lower anterior teeth did not have adequate abutment height for the retention of PFM crowns, crown-lengthening of lower anteriors and posterior passive supraeruption were a part of the treatment plan.

Both diurnal and nocturnal bruxism has been found to be related to extensive tooth wear.[9] Bruxism is most commonly defined as the gnashing and grinding of teeth for nonfunctional purposes. Bruxism may result in mobility, fracture, intrusion, obtrusion, abfraction, opening of contacts, drifting, erosion, abrasion or pulp pathology. Astute questioning of the patient and family members may help in finding out about some of these habits. The fabrication of a hard plastic interocclusal device and making the patients to wear it at night or during the day may be a very useful preventive treatment for many of these patients.[10]

Anterior guidance is one of the “end factors” (with condylar guidance) that ultimately will determine the occlusal anatomy of the finished posterior restorations.[11] If the anterior teeth are badly broken down due to severe tooth wear, most likely the anterior guidance will be less than ideal or perhaps completely absent and so it should be restored first, as posterior restorations constructed under these circumstances would need to have a flat or very shallow occlusal anatomy to avoid interferences on eccentric movements. This would affect the esthetics and possibly the function of the finished restorations. Later construction of the anterior restorations would result in an increased disocclusion of the posterior teeth in eccentric movements, which would allow a sharper, more definitive occlusal anatomy on the posterior teeth.
